# Signaling of free fatty acid receptors 2 and 3 differs in colonic mucosa following selective agonism or coagonism by luminal propionate

**DOI:** 10.1111/nmo.13454

**Published:** 2018-08-23

**Authors:** Iain R. Tough, Sarah Forbes, Helen M. Cox

**Affiliations:** ^1^ King's College London Wolfson Centre for Age‐Related Diseases, Institute of Psychiatry, Psychology & Neuroscience London UK

**Keywords:** enteric submucosal neurons, enteroendocrine L cells, FFA2 and FFA3, human colon, mouse colon, propionate

## Abstract

**Background:**

Propionate exhibits affinity for free fatty acid receptor 2 (FFA2, formerly GPR43) and FFA3 (GPR41). These two G protein‐coupled receptors (GPCRs) are expressed by enteroendocrine L cells that contain anorectic peptide YY (PYY) and glucagon‐like peptide 1 (GLP‐1), while FFA3 is also expressed by enteric neurons. Few studies have investigated the individual roles of FFA2 and FFA3 in propionate's gastrointestinal (GI) effects. Here, we compared FFA2, FFA3, and propionate mucosal responses utilizing selective ligands including an FFA3 antagonist, in mouse and human colonic mucosa.

**Methods:**

Vectorial ion transport was measured in native colonic preparations from normal mouse and human colon with intact submucosal innervation. Endogenous fecal pellet propulsion was monitored in colons isolated from wild‐type (WT) and PYY−/− mice.

**Key Results:**

FFA2 and FFA3 signaling differed significantly. FFA2 agonism involved endogenous L cell‐derived PYY and was glucose dependent, while FFA3 agonism was independent of PYY and glucose, but required submucosal enteric neurons for activity. Tonic FFA3 activity was observed in mouse and human colon mucosa. Apical propionate responses were a combination of FFA2‐PYY mediation and FFA3 neuronal GLP‐1‐ and CGRP‐dependent signaling in mouse ascending colon mucosa. Propionate also slowed WT and PYY−/− colonic transit, and this effect was blocked by a GLP‐1 receptor antagonist.

**Conclusions & Inferences:**

We conclude that luminal propionate costimulates FFA2 and FFA3 pathways, reducing anion secretion and slowing colonic motility; FFA2 via PYY mediation and FFA3 signaling by activation of enteric sensory neurons.

**Abbreviations**
5‐HT: 5‐hydroxytryptamine
AC: ascending colon
ACh: acetylcholine
AR420626: N‐(2,5‐dichlorophenyl)‐4‐(furan‐2‐yl)‐2‐methyl‐5‐oxo‐1,4,5,6,7,8‐hexahydro quinoline‐3‐carboxamide
BIBN4096: 1‐[3,5‐Dibromo‐*N*‐[[4‐(1,4‐dihydro‐2‐oxo‐3(2*H*)‐quinazolinyl) ‐1‐piperidinyl] carbonyl]‐D‐tyrosyl‐L‐lysyl]‐4‐(4‐pyridinyl)‐piperazine
BIBO3304: (*R*)‐*N*‐[[4‐(aminocarbonyl aminomethyl)‐phenyl]methyl]‐N^2^‐(diphenylacetyl)‐argininamide trifluoroacetate)
BIIE0246: (S)‐N2‐[[1‐[2‐[4‐[(R,S)‐5,11‐dihydro‐6(6h)‐oxodibenz[b,e]azepin‐11‐yl]‐1‐piperazinyl]‐2 oxoethyl]cyclopentyl]acetyl]‐N‐[2‐[1,2‐dihydro‐3,5(4H)‐dioxo‐1,2‐diphenyl‐3H‐1,2,4‐triazol‐4‐yl]ethyl]‐argininamide
CCh: carbachol
CGRP: calcitonin gene‐related peptide
Compound 1: (2S,5R)‐5‐(2‐chlorophenyl)‐1‐1(2'‐methoxy‐[1,1'‐biphenyl]‐4‐carbonyl) pyrrolidine‐2‐carboxylic acid
DC: descending colon
DMSO: dimethyl sulfoxide
DPPIVI: dipeptidyl peptidase‐4 inhibitor
ENS: enteric nervous system
Ex(9‐39): exendin(9‐39)
FFA: free fatty acid
FFA2: free fatty acid receptor 2 (formerly known as GPR43)
FFA2−/−: FFA2 knockout mice
FFA3: free fatty acid receptor 3 (formerly GPR41)
FFA3−/−: FFA3 knockout mice
GI: gastrointestinal
GLP‐1: glucagon‐like peptide 1
GPCR: G protein‐coupled receptor
HFD: high‐fat diet
Isc: short‐circuit current
KH: Krebs‐Henseleit
PA: (S)‐2‐(4‐chlorophenyl)‐3, 3‐dimethyl‐N‐(5‐phenylthiazol‐2‐yl)butanamide
PYY(3‐36): peptide YY(3‐36)
PYY: peptide YY
PYY−/−: peptide YY knockout mice
RS39604: 1‐(4‐amino‐5‐chloro‐2‐(3,5 dimethyloxy)benzyloxyphenyl)‐3‐[1‐((2‐methyl sulfonyl‐amino)ethyl)piperidin‐4‐yl]‐1‐propanone hydrochloride
SCFAs: short‐chain fatty acids
SGLT1: sodium‐glucose cotransporter 1
TTX: tetrodotoxin
UGIT: upper gastrointestinal transit
VIP: vasoactive intestinal polypeptide
WT: wild‐type

**Key Points**

- Short chain fatty acid (SCFA) receptors, FFA2 and FFA3, are expressed by enteroendocrine cells, with FFA3 also on enteric neurons. Selective ligands for these receptors have recently become available. We compared FFA2 and FFA3 agonism and propionate responses in gastrointestinal mucosae.
- FFA3 signaling involved enteric neurons and was glucose independent, whereas FFA2 signaling involved PYY and was mucosal derived. Luminal propionate costimulated FFA2 and FFA3 signaling and slowed colonic transit.
- SCFAs coactivate enteric mucosal hormone and neural pathways to modulate gut functions.

## 1 INTRODUCTION

The beneficial effects of dietary fiber are partially due to its microbial metabolism into the short‐chain fatty acids (SCFAs) such as acetate and butyrate, with propionate accounting for ~25% of these fermentation products.^1^ Intracolonic infusion of propionate stimulates the corelease of glucagon‐like peptide‐1 (GLP‐1) and peptide YY (PYY) in rodents^2^, ^3^ and man,^4^ promoting energy metabolism,^5^ and preventing weight gain in overweight subjects.^6^ SCFAs also exhibit a range of gastrointestinal (GI) activities that include epithelial barrier maintenance,^7^ altered motility,^8^, ^9^, ^10^, ^11^ and epithelial ion transport^12^, ^13^, ^14^ that involve PYY and GLP‐1,^15^, ^16^ while butyrate is additionally (and uniquely among SCFAs) an energy source for enterocytes. SCFAs activate several G protein‐coupled receptors (GPCRs) whose functional significance remains obscure primarily because of a lack of selective ligands. GI mucosal signaling is further complicated by the presence of SCFA transporters that readily absorb these anions.^14^ Nevertheless, studies utilizing selective antagonists and agonists have enabled some resolution of two GPCRs, namely free fatty acid 2 (FFA2; formerly GPR43) and FFA3 (GPR41).^15^, ^16^, ^17^ In GI mucosae, FFA2 and FFA3 responses appear to be Gα_q_ linked in L cells^15^, ^16^ while Gα_i/o_ coupling mediates FFA2‐induced ghrelin secretion.^18^ Propionate exhibits a similar affinity for FFA2 and FFA3 (as does acetate), but butyrate preferentially binds FFA3 and another receptor, GPR109A (also known as HCA2).^19^ Notably, FFA2 and FFA3 GI expression patterns differ. In the distal small intestine and colon, FFA2 is expressed predominantly by L cells (that contain PYY and GLP‐1), submucosal leukocytes, and mast cells,^16^, ^20^ while FFA3 is expressed in L cells and enteric neurons in both myenteric and submucosal ganglia.^16^

Selective FFA2 or FFA3 ligands have become commercially available^21^, ^22^, ^23^ and revealed discrete functions for FFA2^15^, ^16^, ^24^ and FFA3.^16^, ^25^ Using a selective FFA2 agonist (named Compound 1), we showed that FFA2 agonism induced PYY, rather than GLP‐1 activity in mouse colonic mucosa, activating anorexigenic pathways without improving glucose tolerance in vivo in lean or obese mice.^24^ The majority of L cell vesicles contain one or the other peptide;^26^ thus, independent release is possible but has not been a common observation to date. In fact, this selective FFA2 agonist suppressed insulin levels in vivo, so we concluded that FFA2 could be a therapeutic target for obesity rather than type 2 diabetes.^24^ In contrast, FFA3 signaling appears to be primarily neural^16^, ^27^ and in the GI tract may involve cholinergic nicotinic (in the rat ascending colon^17^) and 5‐hydroxytryptamine (5‐HT) mechanisms^27^ and possibly vasoactive intestinal polypeptide (VIP^16^), although FFA3 expression has also been observed in human colon epithelia.^28^ Species variations in the signaling bias of FFA2 and FFA3 agonists have presented significant challenges for their translation.^23^ Mindful of this challenge and the availability of selective agonists, our primary aim was to test the hypothesis that FFA3 signaling was predominantly neuronal, while FFA2 signaling was not neuronally mediated, and propionate was a coagonist at FFA2 and FFA3. The FFA3 antagonist, AR399519 (alongside the FFA3 agonist, AR420626^18^) was used to assess the involvement of FFA3 in propionate responses. Additionally, the regional sensitivity to AR420626 was established in mouse GI tract and, importantly, in human colonic mucosa and the endogenous mediators of FFA3 mucosal signaling were compared with FFA2 enteroendocrine signaling in mouse mucosae.^3^, ^15^, ^24^

## 2 MATERIALS AND METHODS

### 2.1 Materials

BIBO3304, BIIE0246, BIBN4096, and phloridzin were purchased from Tocris (Bristol, UK). Stock solutions of BIBO3304, BIIE0246, and BIBN4096 were dissolved in 10% dimethyl sulfoxide (DMSO, at 1 m mol L^−1^) and were stored at −20°C. The FFA2 agonist, PA was purchased from Calbiochem (Watford, UK), peptides were from Cambridge Bioscience (Cambridge, UK) and stock aliquots were stored at −20°C, undergoing one freeze‐thaw cycle only. Tetrodotoxin (TTX) was purchased from Abcam (Cambridge) while all other agents, including sodium propionate, were from Sigma (Poole, UK).

### 2.2 Methods

#### 2.2.1 Mucosal ion transport (short‐circuit current; I_sc_) in vitro

All mice with the C57BL/6‐129/SvJ background strain had free access to standard chow and water. Animals were housed under controlled conditions (12:12 hours light/dark cycle, lights on 07.00 hours, 22 ± 2°C) and their care and experimental procedures complied with the Animals (Scientific procedures) Act 1986.

Mucosal preparations (0.14 cm^2^ exposed areas) were dissected as described previously^29^ and were devoid of overlying smooth muscle and myenteric plexi but retained intact submucosal innervation. In tissue surveys, two adjacent pieces of mucosae from designated GI areas, that is, duodenum, jejunum, terminal ileum, ascending colon (next to the cecal junction, identified as AC1‐AC2), or descending colon (DC2‐DC1, the latter adjacent to the rectum^30^) were prepared from either wild‐type (WT; PYY+/+) or PYY knockout (PYY−/−) mice and bathed in Krebs‐Henseleit buffer (KH; in m mol L^−1^: NaCl 118, KCl 4.7, NaHCO_3_ 25, KH_2_PO_4_ 1.2, MgSO_4_ 1.2, CaCl_2_ 2.5, D‐glucose 11.1, pH 7.4). Mucosae were voltage clamped at 0 mV in Ussing chambers^24^, ^29^, ^30^ and the resultant short‐circuit current (Isc) was allowed to stabilize before drug addition, recording maximum changes in Isc as μA/cm^2^. The FFA2 and FFA3 agonists (PA; named compound 58 in Wang et  al^31^ and AR420626, respectively), FFA3 antagonist AR399519 (abbreviated to AR19 in^18^), or the SCFA, propionate (5 n mol L^−1^) were added apically unless otherwise stated, while all peptides, other antagonists, and TTX (100 n mol L^−1^) were added basolaterally. In studies comparing ascending and descending colonic mucosae, no more than four adjacent sections were prepared from the most proximal or distal regions, respectively. Addition of PA, AR420626, or propionate occurred 5‐10 minutes after VIP (10 or 30 n mol L^−1^; unless otherwise stated) while PYY (10 n mol L^−1^) or exendin 4 (100 n mol L^−1^) responses were obtained at least 25 minutes after the FFA2/FFA3 ligands. Endogenous PYY, GLP‐1, calcitonin gene‐related peptide (CGRP), acetylcholine (ACh), or 5‐HT mediation of FFA2 or FFA3 activities were determined using optimized pretreatments with selective antagonists, that is, the Y1 antagonist (BIBO3304, BIBO; 300 nM) ± Y2 antagonist (BIIE0246, BIIE; 1 µM) ± GLP‐1 antagonist (exendin(9‐39), Ex(9‐39); 1 µ mol L^−1^), or the CGRP antagonist BIBN4096 (10 n mol L^−1^ or 1 µ mol L^−1^, for ascending or descending colon mucosae^30^), atropine (1 µ mol L^−1^), hexamethonium (200 µ mol L^−1^), or the 5‐HT_4_ antagonist RS39604 (1 µ mol L^−1^). After VIP, FFA2 and FFA3 agonist responses were monophasic reductions in Isc, while propionate initiated biphasic changes in Isc; an initial transient increase in Isc (denoted as the 1˚ component) was followed by a slower secondary (2˚) longer lasting reduction in Isc. These Isc response components were analyzed separately.

In glucose sensitivity studies, the colonic mucosae were bathed with KH containing glucose (11.1 m mol L^−1^) on the basolateral side, but mannitol (11.1 m mol L^−1^) replaced glucose in the apical reservoir,^24^ and apical FFA3 agonist or propionate responses were recorded subsequently. As a control, blockade of the Na^+^‐glucose cotransporter 1 (SGLT1) was achieved with apical phloridzin (50 µ mol L^−1^), which reduced Isc levels, but only in the presence of apical glucose.

#### 2.2.2 Human colonic mucosal studies

Colonic specimens were obtained from patients undergoing elective surgery for colonic cancer. Informed consent was obtained from four patients (two males, two females, mean age 53.5 ± 2.0 year) with ethical approval from the Guy's and St Thomas' Hospitals Research Ethics Committee. Mucosae were prepared as described previously^32^, ^33^ and experimental protocols were the same as those described for murine mucosae but without VIP pretreatment. Based on the maximal responses recorded in mouse mucosae, concentrations of 1 µ mol L^−1^ for AR399519 and 3 µ mol L^−1^ for AR420626 (apically) followed by 100 n mol L^−1^ PYY (basolateral) were added to human colon mucosal preparations.

#### 2.2.3 Endogenous fecal pellet propulsion measurement in mouse colon in vitro

Colonic transit of endogenous fecal pellets was measured in vitro by incubating colons (from the caeco‐colonic junction to the rectum) from WT or PYY−/− mice for 20 minutes in KH buffer containing vehicle (H_2_O or 0.1% DMSO), PA or AR420626 (either at 1 µ mol L^−1^), or propionate (5 m mol L^−1^). Pellet propulsion was assessed by taking photographs at *t* = 0 and *t* = 20 min, measuring the mean pellet movement relative to the total colonic length (quoted as a % of colonic transit), as described previously.^29^ Where colons were pretreated with the GLP‐1 antagonist, Ex(9‐39) fecal pellet positions were measured at *t* = 0 and *t* = 20, to measure the effect of GLP‐1 blockade, following which propionate was added to the KH and transit measured after a further 20 minutes (*t* = 40), with control tissues substituting vehicle (H_2_O) for the antagonist. In the rare event that a pellet was excreted during drug incubation periods, then that pellet's movement was excluded from the pooled data.

#### 2.2.4 Data analysis

All data are presented as means ± 1SEM. Analyses were performed using GraphPad Prism v7.03, by Student's *t*test or one‐way ANOVA with Dunnett's or Bonferroni's multiple comparison post hoc tests, as appropriate. When comparing the effect of a pretreatment or the presence/absence of glucose in an adjacent mucosal preparation, the control and experimental agonist responses were compared using Student's *t*test. When more than one pretreatment was compared (eg, after different antagonists but using the same agonist), then one‐way ANOVA with Dunnett's posttest was applied. *P* ≤ 0.05 was statistically significant.

## 3 RESULTS

### 3.1 Mucosal FFA3 agonism involves submucosal neurons, not PYY mechanisms as observed for FFA2 agonism

**Figure 1:**
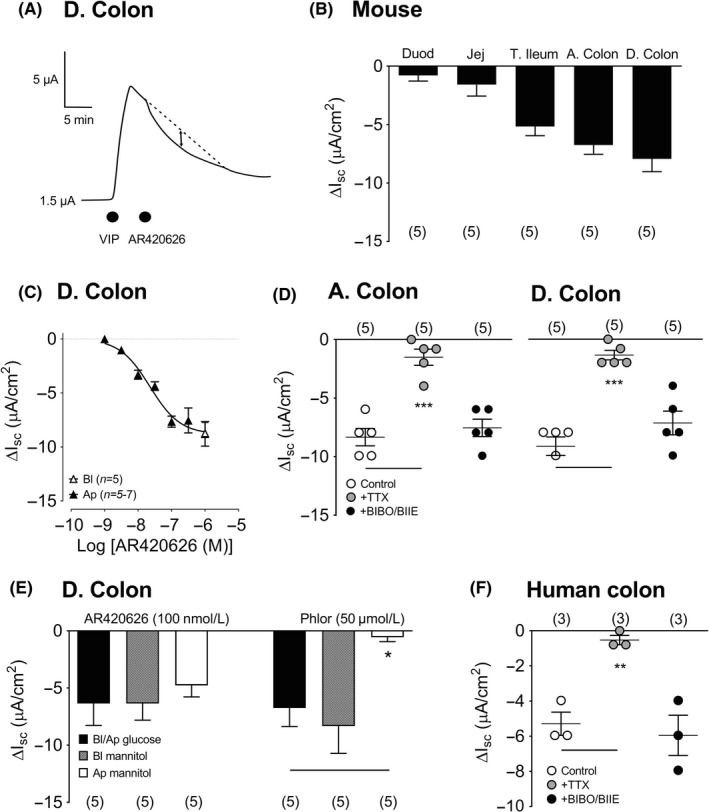
The effect of apical FFA3 agonist AR420626 (100 n mol L^−1^) on VIP pretreated mouse descending colon mucosa (A) and in (B) a comparison of mucosal AR420626 responses in the duodenum (Duod), jejunum (Jej), terminal ileum (T. Ileum), ascending colon (A. Colon), and descending colon (D. Colon). C, A concentration‐response relationship for AR420626 (constructed from single apical additions only) compared with 1 µ mol L^−1^ AR420626 added basolaterally (open triangle) in the mouse D. colon. D, Attenuation of apical FFA3 agonism (1 µ mol L^−1^) following TTX (100 n mol L^−1^) pretreatment but not Y1/Y2 blockade with BIBO3304 and BIIE0246 (+BIBO/BIIE) in mouse A. colon and D. colon mucosae. E, Glucose substitution with mannitol, either basolaterally (Bl) or apically (Ap), had no significant effect on murine FFA3 responses, whereas apical mannitol prevented the effects of SGLT1 inhibitor, phloridzin (Phlor, 50 µ mol L^−1^). F, Apical FFA3 responses (AR420626, 3 µ mol L^−1^) in naive human colon mucosa exhibited sensitivity to TTX, but not to Y1/Y2 antagonism (BIBO/BIIE). Values are the mean ± 1SEM from 3‐7 observations and statistical differences between control and experimental groups are as shown (*P ≤ 0.05, **P ≤ 0.01, ***P ≤ 0.001)

FFA3 activity was monitored using the selective agonist, AR420626. When added apically (after VIP), AR420626 elicited monophasic reductions in Isc (Figure 1A) in mucosal preparations from the small and large intestine (Figure 1B). The greatest FFA3 efficacy was observed in distal regions (terminal ileum, ascending, and descending colon) in contrast with more uniform FFA2 signaling (using the commercially available agonist, PA; 100 n mol L^−1^ apically, Supporting information Figure S1A, B). In the descending colon, apical AR420626 responses were concentration dependent, exhibiting an EC_50_ value of 22.6 n mol L^−1^ (11.3‐45.2 n mol L^−1^) and responses to basolateral AR420626 (1 µ mol L^−1^) were identical to apical responses (Figure 1C). Apical PA concentration‐responses exhibited an EC_50_ of 29.5 n mol L^−1^ (8.8‐94.5 n mol L^−1^) in the ascending colon (Supporting information Figure S1C) and 5.4 n mol L^−1^ (2.1‐14.2 n mol L^−1^) in the descending colon (Supporting information Figure S1D). FFA2 mucosal signaling (Supporting information Figure S1E, F) differed significantly from FFA3 agonism. The latter was abolished by the neurotoxin TTX but was unaffected by pretreatment with PYY‐Y1 and Y2 antagonists, in ascending and descending colon (Figure 1D). This indicates a submucosal neuron‐dependent, PYY‐independent FFA3 mechanism, while FFA2 responses were PYY‐Y1/Y2 mediated and not neuronal in the same colonic regions. TTX alone reduced basal Isc levels (data not shown) indicating the presence of a neurogenic secretory tone, as observed previously in mouse and human colon.^29^, ^33^

Previous studies have revealed that FFA2‐induced GLP‐1 and PYY release from murine L cells was glucose dependent.^15^, ^24^ In contrast, we found that FFA3 responses were insensitive to glucose substitution with mannitol on either mucosal surface (Figure 1E). Predictably, the internal control, phloridzin was only effective when apical glucose was present, representing the blockade of this absorptive Na^+^‐linked mechanism (Figure 1E). Notably, AR420626 mucosal responses in human colon mucosa were also monophasic reductions in Isc and this activity was TTX sensitive, and Y1/Y2 independent (Figure 1F), demonstrating conserved FFA3 mechanisms in human and mouse colonic mucosae.

**Figure 2:**
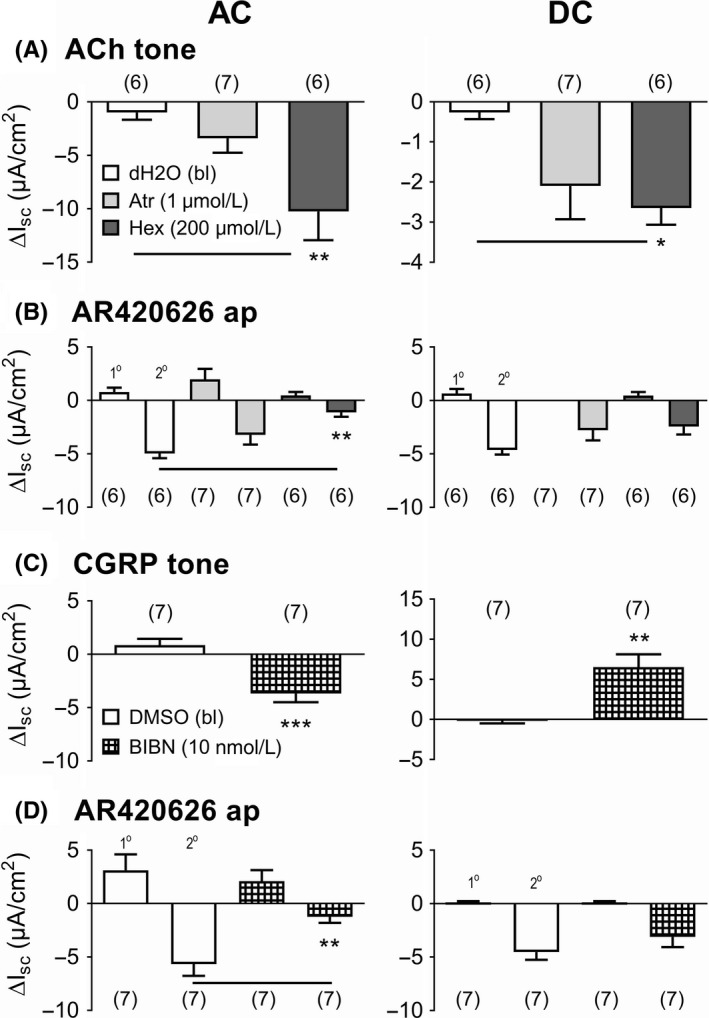
Pretreatment with acetylcholine (ACh) antagonists (atropine (Atr) or hexamethonium (Hex); A, B) or the CGRP antagonist (BIBN4096 (BIBN); C, D) inhibit apical FFA3 agonism in the mouse ascending colon (AC; LHS) but not in the descending colon (DC; RHS). A, Naive murine mucosae (ie, no VIP addition) were pretreated with optimal muscarinic (Atr) or nicotinic antagonism (Hex) at the concentrations shown, revealing consistent cholinergic tone (A). Subsequent biphasic responses to apical AR420626 (in B: 100 n mol L^−1^, identified as 1˚ or 2˚ Isc components) were compared with vehicle controls. C, CGRP antagonism with BIBN4096 (10 n mol L^−1^ or 1 µ mol L^−1^ in AC or DC, respectively^30^) blocked net CGRP tone that differed in murine AC and DC, while subsequent AR420626 (in D; 100 n mol L^−1^, apically) responses were significantly reduced in the ascending colon only (LHS). Note the difference in y axes in A & C. Values are the mean ± 1SEM (from numbers shown in parentheses) and statistical differences between vehicle and experimental groups are as shown (*P ≤ 0.05, **P ≤ 0.01, ***P ≤ 0.001)

FFA3 agonism has been shown to inhibit cholinergic neurotransmission in rat colon mucosa^17^ while in mouse small intestine, FFA3 is colocalized with VIP in neurons located within both the submucosal and myenteric plexi.^16^ We set out to ascertain which neurotransmitters predominantly mediate FFA3 responses but could not pursue VIP‐specific mechanisms, as in our hands, none of the commercially available antagonists block VPAC responses (Cox et  al. unpublished findings). We therefore focussed on cholinergic mechanisms utilizing the muscarinic antagonist, atropine, or nicotinic blocker hexamethonium, which revealed a significant nicotinic tone that was greater in the ascending than the descending colon mucosa (Figure 2A). Only in the proximal colon, hexamethonium significantly inhibited the 2˚ (antisecretory) FFA3 activity of AR420626 (Figure 2B). Muscarinic blockade had no effect on either component of the AR420626 response, although atropine abolished subsequent carbachol (CCh) responses (data not shown) in both regions. We have previously shown that CGRP is coexpressed in cholinergic submucosal neurons in the mouse colon^34^ and so the CGRP antagonist, BIBN4096 was utilized (at the optimal concentrations previously shown to block CGRP activity).^30^ Significant but different net CGRP tonic activity was observed in proximal versus distal colon, as seen previously^30^ (Figure 2C). In vehicle‐treated ascending colon, AR420626 mucosal responses were clearly biphasic (a primary [1˚] increase in Isc followed by a secondary [2˚] Isc decrease [Figure 2D]). CGRP antagonism selectively blocked the 2˚ component of the FFA3 response, but this aspect was unaffected in the descending colon, revealing mechanistic differences in mucosal FFA3 signaling within the mouse colon. Control CGRP (10 n mol L^−1^) or CCh (10 µ mol L^−1^) responses were selectively abolished by BIBN4096 or hexamethonium, in both colonic regions (data not included).

### 3.2 FFA3 antagonism reveals tonic FFA3 activity in mouse and human colonic mucosa

**Figure 3:**
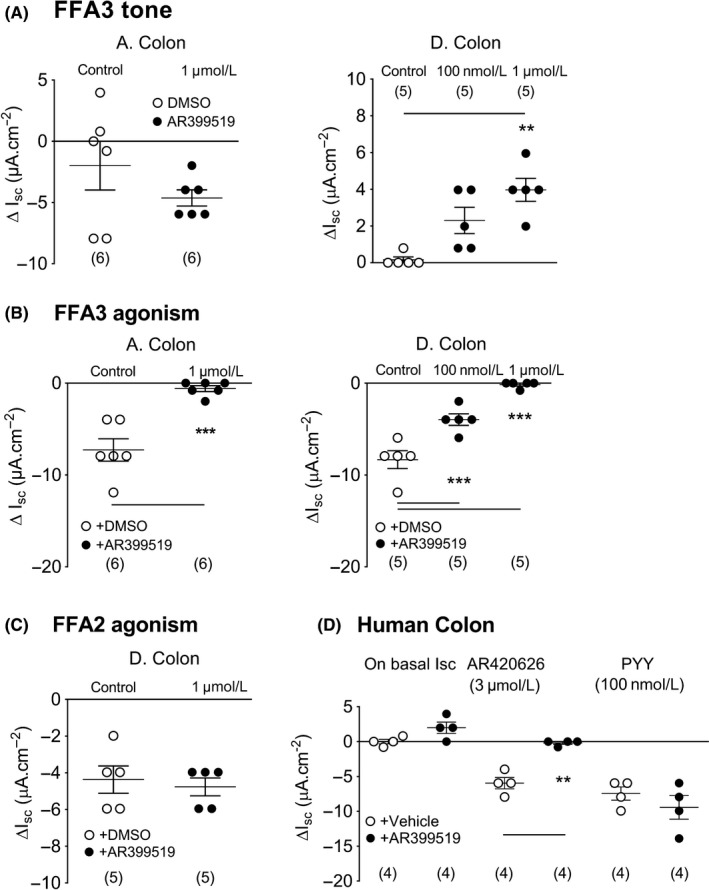
Apical FFA3 antagonism with AR399519 (A) reduced basal Isc in mouse ascending colon (A. Colon) and raised Isc in mouse descending colon (D. Colon). B, The FFA3 antagonist attenuated subsequent FFA3 responses to apical AR420626 (100 n mol L^−1^) in both colonic regions. C, In contrast, FFA2 agonism (100 n mol L^−1^ PA, apical) was unaffected by the FFA3 antagonist in the mouse distal colon. D, Similar increases in basal Isc were observed with antagonist, AR399519 (1 µ mol L^−1^, apical) and inhibition of subsequent AR420626 (3 µ mol L^−1^) responses was observed in human colon, where PYY (100 n mol L^−1^) responses were unaffected. Values are the mean ± 1SEM from 4‐6 observations (as shown) and statistical differences between control and experimental groups are shown (**P ≤ 0.01, ***P ≤ 0.001)

Competitive FFA3 antagonism with AR399519 (applied apically) revealed a degree of tonic FFA3 activity that was antisecretory in the ascending colon (Figure 3A, left histogram) but prosecretory in the descending colon (and with concentration dependence; Figure 3A, right histogram). In both colonic regions, FFA3 agonism was abolished by AR399519 (Figure 3B), while PYY responses were unaffected by the FFA3 antagonist (data not shown). Furthermore, AR399519 had no significant effect upon FFA2 agonism in mouse descending colon (Figure 3C). In human colon, AR399519 revealed tonic mucosal FFA3 activity similar to that observed in mouse descending colon. The FFA3 antagonist also abolished subsequent AR420626 responses and it had no effect upon PYY responses in human colon mucosa (Figure 3D) demonstrating conserved mechanisms of FFA3 signaling in mouse and human colon mucosa.

### 3.3 Apical propionate responses are biphasic and involve endogenous GLP‐1 and PYY‐Y1 and Y2 mechanisms in mouse colon mucosa

**Figure 4:**
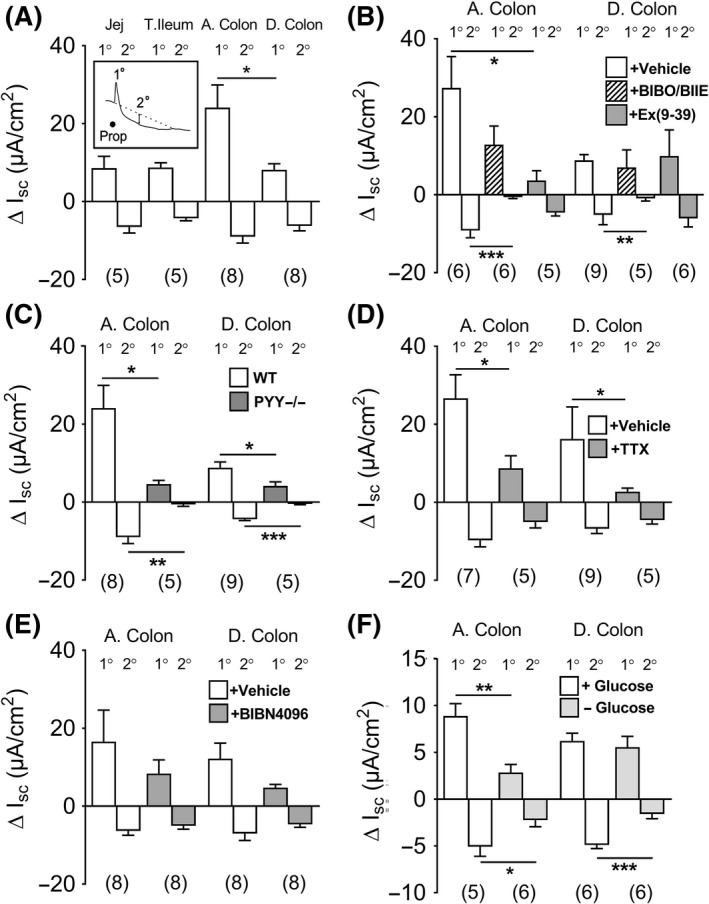
The biphasic primary (1^o^) and secondary (2^o^) changes in Isc following apical propionate (Prop; 5 m mol L^−1^) after VIP (in A) mouse jejunum (Jej), terminal ileum (T. Ileum), ascending colon (A. Colon: and illustrated in the representative response [inset]) and descending colon (D. Colon) after pretreatment with BIBO3304 and BIIE0246 (+BIBO/BIIE), or Ex(9‐39) or vehicle (in B), in PYY−/− mice (C) or TTX or vehicle pretreatment (D) or BIBN4096 (1 µ mol L^−1^) or vehicle (E), and presence/absence of apical glucose (F) in the A. colon and D. colon mucosae from WT mice. Values are the mean ± 1SEM from 5‐9 observations and statistical differences between control and experimental response components are as shown (*P ≤ 0.05, **P ≤ 0.01, ***P ≤ 0.001)

Having established the differences between FFA2 and FFA3 mucosal signaling, we next investigated propionate's acute effects, anticipating a combination of FFA2 and FFA3 agonism. This SCFA (at 5 m mol L^−1^, apically) caused biphasic Isc changes in naive and VIP‐treated preparations from the ascending colon (see the representative response, inset Figure 4A) and descending colon mucosa (Supporting information Figure S2 A and B). To be consistent with our FFA2 and FFA3 studies, we focussed on apical propionate administration and initially surveyed propionate's responses in different GI regions. Propionate consistently evoked biphasic responses in WT jejunum, terminal ileum, ascending and descending colon mucosa, with significantly larger 1˚ increases in Isc in the ascending colon (Figure 4A). Apical propionate responses were slightly, but not significantly larger than their basolateral counterparts (Supporting information Figure S2A), and the biphasic character of propionate effects was consistent along the length of the mouse GI tract. Propionate signaling was more pronounced in the ascending colon compared with descending colon mucosa (Figure 4A, Supporting information Figure S2).

The GLP‐1 antagonist, Ex(9‐39), significantly reduced the propionate 1˚ component in the ascending colon, but it had no effect on the significantly smaller 1˚ component in the descending colon (Figure 4B). The Y1 and Y2 antagonists (BIBO3304 and BIIE0246, respectively) attenuated the 2˚ Isc reduction to propionate in both colonic regions, indicating a consistent PYY‐Y1 and Y2 mechanism for propionate's antisecretory response (Figure 4B). Subsequent responses to exogenous PYY and GLP‐1 agonist exendin 4 were abolished by their respective antagonists (data not shown), as reported previously.^24^ PYY−/− mucosae exhibited similar loss of propionate signaling (Figure 4C) to that seen in WT mucosa treated with Y1 and Y2 antagonists (Figure 4B). Interestingly, both the 1˚ and 2˚ responses to propionate were significantly inhibited in PYY−/− ascending and descending colon (Figure 4C) revealing PYY as a mediator of both aspects of the SCFA response. Propionate's 1˚ response was also TTX sensitive in ascending and descending colon (Figure 4D), highlighting a consistent neural involvement. The propionate's 1˚ response was also partially inhibited by the CGRP antagonist (BIBN4096), but this did not reach statistical significance in either colonic area (Figure 4E). Taken together, endogenous PYY appears to provide both a neural Y2 mechanism and an epithelial Y1 antisecretory contribution to propionate's 1˚ and 2˚ responses, respectively,^29^ while GLP‐1 (Ex(9‐39)‐sensitive) and possibly also neural CGRP appear to contribute to propionate's 1˚ response.

The removal of apical glucose significantly reduced the 1˚ propionate's response in ascending colon and it also significantly inhibited the PYY‐mediated 2˚ component in both colonic regions (Figure 4F). This glucose sensitivity is most likely FFA2 mediated, that is, L cell derived, since selective FFA2 agonism in mucosal preparations (with PA or Cpd1^24^) is entirely glucose dependent. In contrast, propionate's 1˚ response in the descending colon was glucose independent, and notably, this component was not GLP‐1 mediated.

### 3.4 FFA3 antagonism inhibits propionate responses in mouse colon mucosae

**Figure 5:**
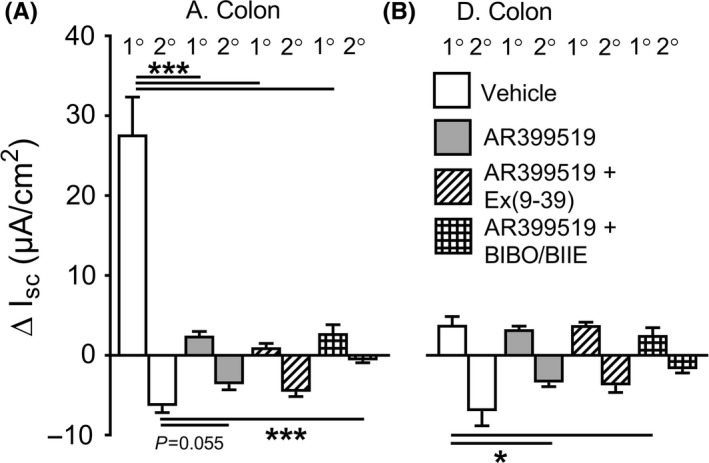
The biphasic changes in Isc with apical propionate (5 m mol L^−1^) in WT mouse ascending colon (A. Colon in A) and descending colon (D. Colon in B). Pretreatment with FFA3 antagonist AR399519 alone (1 µ mol L^−1^) or in combination with Ex(9‐39) (1 µ mol L^−1^) or AR399519 and Y1/Y2 antagonists (+BIBO/BIIE), compared with propionate responses after vehicle controls. Values are the mean ± 1SEM from 5‐6 observations and statistical differences are as shown (*P ≤ 0.05, ***P ≤ 0.001)

FFA3 antagonism with AR399519 revealed a difference in FFA3 tonic activity in the mouse ascending versus the descending colon (Figure 3A). In further support of this observation, the presence of AR399519 virtually abolished the 1˚ responses to propionate in the ascending colon (Figure 5A) but had no effect on the smaller 1˚ response in the descending colon (Figure 5B). The 1˚ component of propionate's response is predominantly neuronal (Figure 4D), so we infer that FFA3 is most likely to be present on submucosal secretory neurons that innervate the colonic epithelium. Blockade of FFA3 with AR399519 also attenuated the 2˚ response to propionate, significantly so in the descending colon, revealing a FFA3 contribution to propionate's antisecretory action, particularly in the distal colon (Figure 5B).

The combination of FFA3 blockade (with AR399519) and either the GLP‐1 antagonist, Ex(9‐39), or Y1 and Y2 antagonists was tested to establish whether endogenous GLP‐1 and/or PYY mediate the residual propionate response, that is, in a FFA3‐independent manner. Ex(9‐39) did not significantly alter either the component of propionate's mucosal response in the presence of FFA3 blockade in ascending colon (Figure 5A). In contrast, propionate's 2˚ antisecretory response was significantly attenuated by the combination of FFA3 antagonism and Y1 and Y2 blockers together (Figure 5A, B). Therefore, a FFA3‐independent but nevertheless L cell‐derived PYY mechanism is likely to be present along the length of the mouse colon, endogenous PYY acting on epithelial Y1 and neural Y2 receptors in response to apical administration of propionate.

The relative resistance of the small propionate's 1˚ responses in descending colon mucosa to antagonists prompted us to investigate these predominantly neural, glucose independent increases in Isc. Previous studies have shown that FFA3 colocalizes with 5‐HT‐containing enterochromaffin (EC) cells within the mucosa,^35^, ^36^ so we utilized the 5‐HT_4_ antagonist, RS39604 to block endogenous 5‐HT signaling. RS39604 alone revealed minimal tonic 5‐HT_4_ activity compared with significant levels of PYY‐Y1/Y2 tone (Supporting information Figure S3A). Neither 1˚ nor 2˚ components of the apical propionate response were altered by 5‐HT_4_ antagonism (Supporting information Figure S3B) while subsequent exogenous 5‐HT responses were abolished (Supporting information Figure S3C). As seen previously, Y1 and Y2 antagonism abolished the 2˚ propionate's response as well as subsequent exogenous PYY responses (Supporting information Figure S3D). Thus, propionate elicits a secretory, neural FFA3‐mediated effect that is glucose insensitive and appears to be 5‐HT independent.

### 3.5 FFA2 and FFA3 agonists slow fecal pellet propulsion in vitro

**Figure 6:**
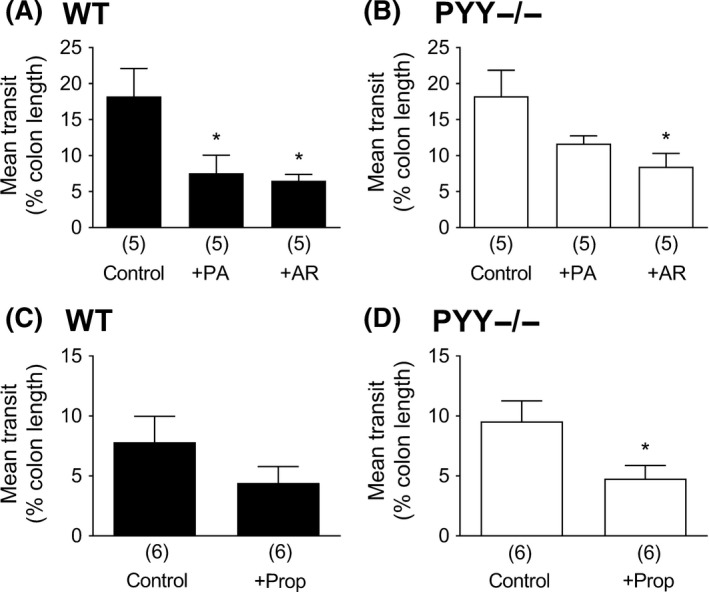
Fecal pellet transit in WT and PYY‐/‐ mouse colon in vitro, in vehicle controls (0.1% DMSO in A & B; H_2_O in C & D), or FFA2 agonist PA (1 µ mol L^−1^, +PA), FFA3 agonist AR420626 (1 µ mol L^−1^, +AR), or, in C & D, propionate (+Prop; 5 m mol L^−1^)‐treated tissue. Values are the mean + 1SEM (from numbers shown in parentheses) with statistical differences between drug‐treated and control groups as shown (*P ≤ 0.05)

In vitro studies have shown previously that L cell‐derived PYY mediates free fatty acid receptor FFA1 and FFA4 activation in mucosal preparations and that selective FFA1 and FFA4 agonists also slow colonic transit in vitro and in vivo.^37^ Having uncovered divergent FFA2 and FFA3 signaling mechanisms in the mouse colon mucosa, we compared FFA2 and FFA3 modulation of endogenous fecal pellet propulsion and with propionate's activity. Individual FFA2 or FFA3 agonism slowed motility significantly in WT colon to similar degrees (Figure 6A). The same agonist concentrations also reduced motility in PYY−/− colon, but PA (the FFA2 agonist) was slightly less effective, while the FFA3 antimotility effect was significant (Figure 6B). Propionate also slowed pellet propulsion to a similar degree in both genotypes (Figure 6C, D), significantly so in PYY−/− colon, indicating that PYY is not the sole mediator of this SCFA's antimotility effects in the mouse colon.

**Figure 7:**
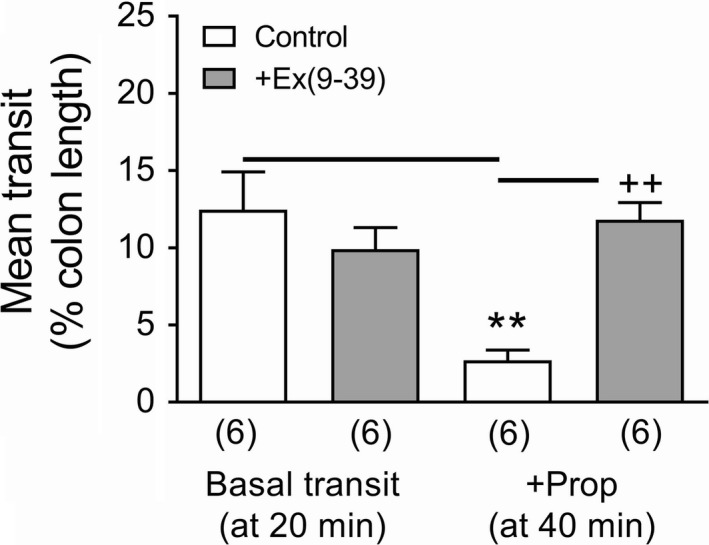
Fecal pellet transit in PYY−/− mouse colon in vitro was unaffected by 20‐min incubation with 1 µ mol L^−1^ Ex(9‐39) compared with vehicle controls (H_2_O); however, a further 20‐min incubation with propionate (+Prop; 5 m mol L^−1^) attenuated basal transit in control colons but not in Ex(9‐39) pretreated colons. Values are the mean + 1SEM (from number shown in parenthesis). **P ≤ 0.01 compared with control at 20 min; **++** *P *≤ 0.01 compared with control +Prop at 40 min

We investigated the inhibitory effect of propionate in PYY−/− colon further by pretreating the colon with the GLP‐1R antagonist, Ex(9‐39) as GLP‐1 was considered to be the most likely mediator.^15^ Ex(9‐39) per se had no effect on fecal pellet transit; however, after 20‐minute incubation with propionate, Ex(9‐39) blocked the SCFA's inhibitory effect (Figure 7).

## 4 DISCUSSION

### 4.1 Differences between FFA2‐ and FFA3‐specific mucosal activities but common antimotility effects

Small molecule agonists selective for either FFA2 or FFA3 elicited monophasic antisecretory responses along the length of the mouse GI tract and in human colon mucosa. Their apical and basolateral responses exhibited similar time courses and efficacies (as observed previously for FFA2 agonism in the descending colon^24^) indicating that these receptors are probably located on both epithelial domains, that is, positioned to sense SCFAs in the gut lumen and the lamina propria. However, given the difference between circulating (10‐100 µ mol L^−1^) and luminal SCFA levels (50‐100 m mol L^−1^),^10^ it is likely that SCFAs are sensed by basolateral receptors as their affinities for SCFAs are within the plasma concentration range.^19^, ^38^

Apical and basolateral propionate responses were also similar but in contrast with FFA2 and FFA3 responses, the changes in Isc were biphasic and markedly so in the ascending colon. Other rodent studies have observed biphasic (sometimes triphasic) changes in Isc to apical propionate, for example, in guinea pig distal colon with 50 m mol L^−1^ propionate.^39^ In rat colon mucosa, propionate Isc responses were only observed after apical SCFA addition and interestingly involved submucosal cholinergic neurons^12^ implicating transepithelial movement and a basolateral mechanism reminiscent of the neural FFA3 response we observed in the present study. The absence of latency together with the similarity in propionate response kinetics supports the presence of receptors on apical and basolateral surfaces.

**Figure 8:**
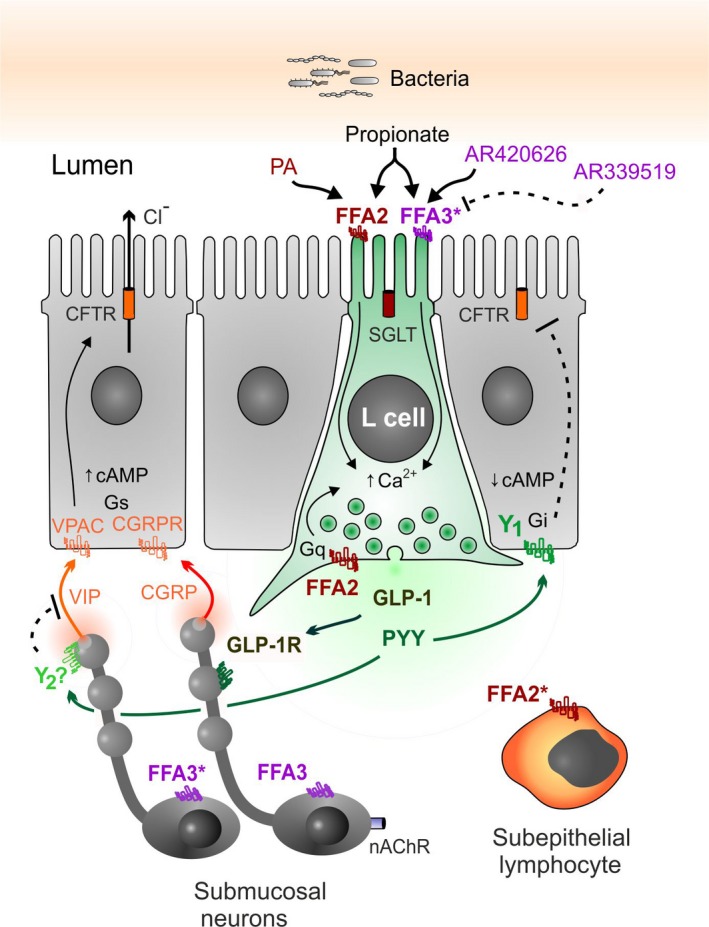
Working model showing the cellular locations of FFA2 and FFA3 activated by lumenal propionate or specific agonists, plus likely peptide mediators and the simplest neural circuitry, supported by our functional data in mouse ascending colon. Additionally, the mechanisms identified by Nøhr et  al^16^ are highlighted by asterisks (*), that is, VIP‐containing submucosal neurons and L cell expression of FFA3, also subepithelial lymphocyte FFA2 expression. In the ascending and descending colon of mouse and human colon, FFA2 is present on apical and basolateral L cell membranes, while FFA3 is predominantly neuronal, potentially on cholinergic, CGRP‐containing submucosal neurons. In mouse ascending colon, L cell‐derived PYY and GLP‐1 partially mediate apical propionate responses, while PYY predominantly mediates FFA2 agonism.^24^

However, the cellular mechanisms of FFA2 and FFA3 selective signaling and their glucose dependence differed markedly, as hypothesized. FFA2 agonism involved L cell‐derived PYY and this mechanism was glucose dependent^24^ and enteric neuron independent, while FFA3 signaling was glucose independent and involved submucosal cholinergic neurotransmission (nicotinic, predominantly) in combination with CGRP (Figure 8). Notably, the neural FFA3 activity was also observed in human colon mucosa. Nohr et  al^16^ described FFA3 expression in submucosal VIP‐positive neurons of murine small intestine, that are likely to be secretomotor/vasodilator in character. However, functional confirmation of VIP's involvement was not possible in the present study due to the current lack of suitably selective VIP receptor antagonists (Cox & Tough, unpublished findings).

Previously we established that selective FFA2 agonism involved endogenous PYY antisecretory and antimotility responses, alongside a suppression of food intake that lead to a reduction in body weight in WT mice, and these activities were absent in FFA2−/− mice or their tissues.^24^ We were unable to detect significant GLP‐1 involvement using this first‐in‐class FFA2 agonist; a finding that was corroborated using the commercially available FFA2 agonist, PA in the present study. However, GLP‐1 release occurs with FFA2 agonists or SCFA administration to colonic crypt cultures and this is a glucose‐dependent mechanism.^3^, ^15^, ^16^, ^40^ In native mucosal preparations, secreted GLP‐1 is probably inactivated rapidly^24^ and its signaling capacity may also be compromised by relatively low levels of GLP‐1 receptor expression. Differential PYY and GLP‐1 signaling is possible as the majority of resolvable L cell vesicles (observed by high‐resolution confocal microscopy) contain PYY or GLP‐1^26^ rather than peptide copackaging as indicated by early ultrastructural studies.^41^ Hence, preferential FFA2‐PYY signaling could be functionally significant, and its amplification by SCFA‐enhanced PYY transcription also holds therapeutic potential.^42^ The neural FFA3 activity that we observed here in mouse and human colon also differs markedly from FFA1 and FFA4 agonism,^37^ GPR119 activity^43^, ^44^ as well as other L cell sensing mechanisms that all involve endogenous PYY and/or GLP‐1 and require glucose.^45^, ^46^ In addition to its neural expression, FFA3 is to some extent expressed by L cells, for example, in mouse proximal colon,^47^ and the same FFA3 agonist that we used (AR420626, but at ≥100x the concentration) stimulated GLP‐1 release from colonic crypt preparations, although with much lower efficacy than propionate.^16^ Taken together, it appears that neither PYY nor GLP‐1 is involved in acute FFA3 activity in mouse or human colonic mucosa and that neural FFA3 pathways predominate over FFA3 L cell signaling in these native tissues.

The FFA3 antagonist AR399519 revealed a degree of prosecretory tone in mouse and human distal colon. Ascending colon mucosa in contrast exhibited antisecretory FFA3 tone. This regional difference was also seen for CGRP tonic activity, implicating a functional link between FFA3 and sensory CGRP‐mediated neural activity. Tonic cholinergic transmission in contrast (as revealed by nicotinic blockade) was consistently prosecretory in mouse colon. Both the CGRP antagonist BIBN4096 and hexamethonium attenuated FFA3‐induced reductions in Isc (the 2˚ component, Figure 2), significantly so in ascending colon, and we conclude that FFA3 agonism involves submucosal CGRP and cholinergic neurotransmission in this region (Figure 8). Previously, we found that all cholinergic submucous plexus neurons contain CGRP (in the distal colon^34^). If these neurons contribute to the tonic activity we observed, then corelease ACh and CGRP may occur and net epithelial anion secretion would result. The sensitivity of FFA3 agonism to cholinergic and CGRP blockade is suggestive that FFA3 agonists (potentially via neural FFA3‐G_i_‐, or GIRK coupling) may inhibit sensory submucous neurons causing hyperpolarization and enteric reflex inhibition that would slow motility, as observed for AR420626 in the colon. The neural mechanisms involved in FFA3 agonism in mouse descending colon were more complex; nicotinic and CGRP blockers were inhibitory here, but not significantly so. It is possible that FFA3 modulation of secretomotor VIP neurons^16^ also reduces VIP release and therefore attenuates VIP‐mediated epithelial hypersecretion, but selective VIP antagonists are currently unavailable to test this possibility.

Maximal FFA2 agonism slowed colonic motility and FFA3 activation retarded motility to a similar degree in WT colon. However, the FFA2 response lost significance in PYY−/− colon, in contrast with FFA3 agonism, which remained significant. We conclude that PYY contributes more to FFA2 agonism than to FFA3's antimotility activity where additional neural and/or other mediator(s), for example, GLP‐1, may be involved. Propionate also slowed colonic motility as expected, but this was not PYY dependent. This SCFA is known to retard colonic transit in mouse and rat models via a combination of PYY and GLP‐1 mechanisms.^3^, ^9^, ^11^, ^15^ We confirmed the involvement of GLP‐1 in propionate's antimotility effect in PYY−/− colon using the GLP‐1 antagonist Ex(9‐39). Thus, propionate slows mouse colonic motility via a combination of endogenous PYY and GLP‐1 mechanisms.

### 4.2 Propionate coactivates FFA2 and FFA3 with additional electrogenic epithelial mechanisms

Mucosal responses to apical propionate were a combination of PYY and GLP‐1 signaling, and this biphasic electrogenic response was consistent and contrasted with the monophasic FFA2 or FFA3 responses in the same mucosal areas. GLP‐1 predominantly mediated the 1˚ component of propionate's response in the mouse ascending colon. Previously, we observed that apical acetate or propionate induced biphasic Isc changes in WT colon mucosa and the slower (2˚) antisecretory component was absent from FFA2−/− mucosa^24^ (Supporting information Data S1). In the present study, we confirmed that this 2˚ aspect was solely PYY mediated as it was abolished by a combination of Y1 and Y2 antagonists in ascending and descending colon mucosae. L cell FFA2, therefore, contributes to the acute propionate response (Figure 8) and this specific activity was glucose sensitive, in agreement with our previous study investigating PYY‐mediated mechanisms,^24^ and the increased PYY and GLP‐1 release observed in vivo to a number of luminal stimuli including propionate.^48^ Additionally, in the ascending colon, the GLP‐1‐mediated 1˚ response to propionate was also glucose dependent. Interestingly, this 1˚ propionate response was abolished by FFA3 antagonism, revealing a SCFA‐stimulated FFA3/GLP‐1 signal that was most likely neuronal as TTX also blocked this activity. Indeed, FFA3 antagonism on baseline revealed antisecretory tone in the ascending colon, and secretory tone in the descending colon, highlighting tonic FFA3 activities with opposing outcomes on mucosal secretion. However, as selective FFA3 agonism did not elicit an increase in Isc in any preparations and we suggest that this may be because the small molecule is not transported across the mucosa and cannot readily access neuronal FFA3. FFA3 blockade also inhibited the 2^o^ propionate response (significantly so in the descending colon) as did Y1/Y2 antagonists, implicating the involvement of endogenous PYY in this glucose‐sensitive component. We, therefore, conclude that the 2^o^ propionate response is likely to be a combination of FFA2 and FFA3 agonism. In the descending colon, the small 1˚ increase in Isc to propionate was significantly inhibited by TTX (Figure 4D) and blunted in PYY−/− tissue (Figure 4C), indicating a neural Y2 mechanism; however, the combination of Y1 and Y2 blockade was lost in the presence of FFA3 blockade indicating that Y2 and FFA3 may be expressed by the same neurons (Figure 8). The residual 1˚ electrogenic response to apical propionate was not 5‐HT‐mediated despite evidence to the contrary^35^, ^36^ and we propose that if endogenous 5‐HT is being released by propionate, then this is transient and much less efficacious than the pharmacologically distinct peptide and neural colonic pathways stimulated by the SCFA. Instead, we suggest that the residual 1˚ component may be a consequence of Na^+^‐coupled propionate absorption via the apical monocarboxylic acid transporter, slc5a8,^49^, ^50^ but we did not investigate this minor electrogenic response further.

Taken together, our findings provide novel functional insights into discrete colonic FFA2 and FFA3‐mediated pathways coactivated acutely by luminal propionate. The data fit with the hypothesis that FFA3 activity is predominantly neuronal while FFA2 signaling is primarily L cell derived. The involvement of FFA2‐induced endogenous PYY and GLP‐1 release^48^ and inhibitory neuronal (FFA3) SCFA mechanisms potentially underpin clinical observations showing that an increase in dietary fiber or intracolonic delivery of propionate elevates postprandial PYY and GLP‐1 levels, reducing energy intake and longer term weight gain in overweight adults,^6^ and promoting energy metabolism.^5^ Based on these findings, we suggest that targeting FFA2 and FFA3 together may offer additional therapeutic potential for the treatment of obesity and type 2 diabetes.

## CONFLICT OF INTEREST

The authors have no competing interests.

## References

[1] den Besten G , van Eunen K , Groen AK , et al. Short chain fatty acids in the interplay between diet, gut microbiota, and host energy. J Lipid Res. 2013;54:2325‐2340. 23821742 10.1194/jlr.R036012 PMC3735932

[2] Plaisancié P , Dumoulin V , Chaivialle J‐A , et al. Luminal peptide YY‐releasing factors in the isolated vascularly perfused rat colon. J Endocrinol. 1996;151:421‐429. 8994387 10.1677/joe.0.1510421

[3] Psichas A , Sleeth ML , Murphy KG , et al. The short chain fatty acid propionate stimulates GLP‐1 and PYY secretion via free fatty acid receptor 2 in rodents. Int J Obes. 2015;39:424‐429. 10.1038/ijo.2014.153 PMC4356745 25109781

[4] Freeland KR , Wolever T . Acute effects of intravenous and rectal acetate on glucagon‐like peptide‐1, peptide YY, ghrelin, adiponectin and tumour necrosis factor‐alpha. Br J Nutr. 2010;103:460‐466. 19818198 10.1017/S0007114509991863

[5] Canfora EE , van der Beek CM , Jocken J , et al. Colonic infusions of short‐chain fatty acid mixtures promote energy metabolism in overweight/obese men: a randomized crossover trial. Sci Rep. 2017;7:2360‐2372. 28539646 10.1038/s41598-017-02546-x PMC5443817

[6] Chambers ES , Viardot A , Psichas A , et al. Effects of targetted delivery of propionate to the human colon on appetite regulation, body weight maintenance and adiposity in overweight adults. Gut. 2015;64:1744‐1754. 25500202 10.1136/gutjnl-2014-307913 PMC4680171

[7] Tappenden KA , McBurney MI . Systemic short‐chain fatty acids rapidly alter gastrointestinal structure, function and expression of early response genes. Dig Dis Sci. 1998;43:1526‐1536. 9690391 10.1023/a:1018819032620

[8] Yajima T . Contractile effect of short‐chain fatty acids on the isolated colon of the rat. J Physiol. 1985;368:667‐678. 2867220 10.1113/jphysiol.1985.sp015882 PMC1192621

[9] Cherbut C , Ferrier L , Roze C , et al. Short‐chain fatty acids modify colonic motility through nerves and polypeptide YY release in the rat. Am J Physiol. 1998;275:G1415‐G1422. 9843779 10.1152/ajpgi.1998.275.6.G1415

[10] Dass NB , John AK , Crumbley CW , et al. The relationship between the effects of short chain fatty acids on intestinal motility in vitro and GPR43 receptor activation. Neurogastroenterol Motil. 2007;19:66‐74. 17187590 10.1111/j.1365-2982.2006.00853.x

[11] Wichmann A , Allahyar A , Greiner TU , et al. Microbial modulation of energy availability in the colon regulates intestinal transit. Cell Host Microbe. 2013;14:582‐590. 24237703 10.1016/j.chom.2013.09.012

[12] Yajima T . Luminal propionate‐induced secretory response in the rat distal colon in vitro. J Physiol. 1988;403:559‐575. 2473196 10.1113/jphysiol.1988.sp017264 PMC1190728

[13] Dagher PC , Egnor RW , Taglietta‐Kohlbrecher A , Charney AN . Short‐chain fatty acids inhibit cAMP‐mediated chloride secretion in rat colon. Am J Physiol. 1996;271:C1853‐C1860. 8997185 10.1152/ajpcell.1996.271.6.C1853

[14] Binder HJ . Role of colonic short‐chain fatty acid transport in diarrhea. Ann Rev Physiol. 2010;72:297‐313. 20148677 10.1146/annurev-physiol-021909-135817

[15] Tolhurst G , Heffron H , Lam YS , et al. Short‐chain fatty acids stimulate glucagon‐like peptide‐1 secretion via the G‐protein‐coupled receptor FFAR2. Diabetes. 2012;61:364‐371. 22190648 10.2337/db11-1019 PMC3266401

[16] Nøhr MK , Pedersen MH , Gille A , et al. GPR41/FFAR3 and GPR43/FFAR2 as cosensors for SCFAs in enteroendocrine cells vs FFAR3 in enteric neurons and FFAR2 in enteric leukocytes. Endocrinol. 2013;154:3552‐3564. 10.1210/en.2013-1142 23885020

[17] Kaji I , Akiba Y , Konno K , et al. Neural FFA3 activation inversely regulates anion secretion evoked by nicotinic ACh receptor activation in rat proximal colon. J Physiol. 2016;594:3339‐3352. 26854275 10.1113/JP271441 PMC4908031

[18] Engelstoft MS , Park W , Sakata I , et al. Seven transmembrane G protein‐coupled receptor repertoire of gastric ghrelin cells. Mol Metabol. 2013;2:376‐392. 10.1016/j.molmet.2013.08.006 PMC3854997 24327954

[19] Husted AS , Trauelsen M , Rudenko O , et al. GPCR‐mediated signaling of metabolites. Cell Metab. 2017;25:777‐796. 28380372 10.1016/j.cmet.2017.03.008

[20] Karaki S , Mitsui R , Hayashi H , et al. Short‐chain fatty acid receptor, GPR43 is expressed by enteroendocrine cells and mucosal mast cells in rat intestine. Cell Tiss Res. 2006;324:353‐360. 10.1007/s00441-005-0140-x 16453106

[21] Ulven T . Short‐chain free fatty acid receptors FFA2/GPR43 and FFA3/GPR41 as new potential therapeutic targets. Front Endocrinol. 2012;3:111 10.3389/fendo.2012.00111 PMC3462324 23060857

[22] Bolognini D , Tobin AB , Milligan G , Moss CE . The pharmacology and function of receptors for short‐chain fatty acids. Mol Pharmacol. 2016;290:18915‐18931. 10.1124/mol.115.102301 26719580

[23] Milligan G , Shimpukade B , Ulven T , Hudson BD . Complex pharmacology of free fatty acid receptors. Chem Rev. 2017;117:67‐110. 27299848 10.1021/acs.chemrev.6b00056

[24] Forbes S , Stafford S , Coope G , et al. Selective FFA2 agonism appears to act via intestinal PYY to reduce transit and food intake but does not improve glucose tolerance in mouse models. Diabetes. 2015;64:3763‐3771. 26239054 10.2337/db15-0481

[25] Nøhr MK , Egerod KL , Christiansen SH , et al. Expression of the short chain fatty acid receptor GPR41/FFAR3 in autonomic and somatic sensory ganglia. Neurosci. 2015;290:126‐137. 10.1016/j.neuroscience.2015.01.040 25637492

[26] Cho H‐J , Robinson ES , Rivera LR , et al. Glucagon‐like peptide 1 and peptide YY are in separate storage organelles in enteroendocrine cells. Cell Tissue Res. 2014;357:63‐69. 24842049 10.1007/s00441-014-1886-9

[27] Kaji I , Akiba Y , Furuyama T , et al. Free fatty acid receptor 3 activation suppresses neurogenic motility in rat proximal colon. Neurogastroenterol Motil. 2018;30:e13157. 10.1111/nmo.13157 PMC5739952 28714277

[28] Tazoe H , Otomo Y , Karaki S , et al. Expression of short‐chain fatty acid receptor GPR41 in the human colon. Biochem Res. 2009;30:149‐156. 10.2220/biomedres.30.149 19574715

[29] Tough IR , Forbes S , Tolhurst R , et al. Endogenous peptide YY and neuropeptide Y inhibit colonic ion transport, contractility and transit differentially via Y_1_ and Y_2_ receptors. Br J Pharmacol. 2011;164:66‐79. 10.1111/j.1476-5381.2011.01401.x PMC3188896 21457230

[30] Tough IR , Moodaley R , Cox HM . Mucosal GLP‐1 responses are mediated by CGRP in the mouse colon and both peptide responses are area‐specific. Neurogastroenterol Motil. 2018;30:e‐13149. 10.1111/nmo.13149 28695626

[31] Wang Y , Jiao X , Kayser F , et al. The first synthetic agonists of FFA2: discovery and SAR of phenylacetamides as allosteric modulators. Bioorg Med Chem Lett. 2010;20:493‐498. 20005104 10.1016/j.bmcl.2009.11.112

[32] Cox HM , Tough IR . Neuropeptide Y, Y1, Y2 and Y4 receptors mediate Y agonist responses in isolated human colon mucosa. Br J Pharmacol. 2002;135:1505‐1512. 11906964 10.1038/sj.bjp.0704604 PMC1573267

[33] Hyland NP , Sjøberg F , Tough IR , et al. Functional consequences of neuropeptide Y Y2 receptor knockout and Y2 antagonism in mouse and human colonic tissues. Br J Pharmacol. 2003;139:863‐871. 12813010 10.1038/sj.bjp.0705298 PMC1573894

[34] Foong J , Tough IR , Cox HM , Bornstein JC . Properties of cholinergic and non‐cholinergic submucosal neurons along the mouse colon. J Physiol. 2014;592:777‐793. 24344165 10.1113/jphysiol.2013.265686 PMC3934714

[35] Tazoe H , Otomoto Y , Kaji I , et al. Roles of short‐chain fatty acids receptors, GPR41 and GPR43 on colonic functions. J Physiol Pharmacol. 2008;59:251‐262. 18812643

[36] Martin AM , Lumsden AL , Young RL , et al. The nutrient‐sensing repertoires of mouse enterochromaffin cells differ between duodenum and colon. Neurogastroenterol Motil. 2017;29:e13046. 10.1111/nmo.13046 28251760

[37] Moodaley R , Smith DM , Tough IR , et al. Agonism of free fatty acid receptors 1 and 4 generates peptide YY‐mediated inhibitory responses in mouse colon. Br J Pharmacol. 2017;174:4508‐4522. 28971469 10.1111/bph.14054 PMC5715575

[38] Cummings JH , Pomare EW , Branch WJ , Naylor C , Macfarlane GT . Short chain fatty acids in human large intestine, portal, hepatic and venous blood. Gut. 1987;28:1221‐1227. 3678950 10.1136/gut.28.10.1221 PMC1433442

[39] Karaki S , Kuwahara A . Propionate‐induced epithelial K^+^ and Cl^−^/HCO_3_ ^−^ secretion and free fatty acid receptor 2 (FFA2, GPR43) expression in the guinea pig distal colon. Pflugers Arch. 2011;461:141‐152. 20945073 10.1007/s00424-010-0889-y

[40] Petersen N , Reimann F , Bartfeld S , et al. Generation of L cells in mouse and human small intestinal organoids. Diabetes. 2011;63:410‐420. 10.2337/db13-0991 PMC4306716 24130334

[41] Bottcher G , Alumets J , Håkanson R , Sundler F . Co‐existence of glicentin and peptide YY in colorectal L‐cells in cat and man. An electron microscopic study. Regul Pept. 1986;13:283‐291. 3754646 10.1016/0167-0115(86)90046-7

[42] Larraufie P , Martin‐Gallausiaux P , Lapaque N , et al. SCFAs strongly stimulate PYY production in human enteroendocrine cells. Sci Rep. 2018;8:1207‐1216. 29311617 10.1038/s41598-017-18259-0 PMC5758799

[43] Cox HM , Tough IR , Woolston A‐M , et al. Peptide YY is critical for acylethanolamine receptor Gpr119‐induced activation of gastrointestinal mucosal responses. Cell Metab. 2010;11:532‐542. 20519124 10.1016/j.cmet.2010.04.014 PMC2890049

[44] Patel S , Mace OJ , Tough IR , et al. Gastrointestinal hormonal responses on GPR119 activation in lean and diseased rodent models of type 2 diabetes. Int J Obes. 2014;38:1365‐1373. 10.1038/ijo.2014.10 24451185

[45] Joshi S , Tough IR , Cox HM . Endogenous PYY and GLP‐1 mediate L‐glutamine responses in intestinal mucosa. Br J Pharmacol. 2013;170:1092‐1101. 23992397 10.1111/bph.12352 PMC3902494

[46] Alamshah A , McGavigan AK , Spreckley E , et al. L‐Arginine promotes gut hormone release and reduces food intake in rodents. Diab Obes Metab. 2016;18:508‐518. 10.1111/dom.12644 PMC4982043 26863991

[47] Symonds EL , Peiris M , Page AJ , et al. Mechanisms of activation of mouse and human enteroendocrine cells by nutrients. Gut. 2015;64:618‐626. 25015642 10.1136/gutjnl-2014-306834 PMC4392230

[48] Mace OJ , Schindler M , Patel S . The regulation of K‐ and L‐cell activity by GLUT2 and the calcium‐sensing receptor CasR in rat small intestine. J Physiol. 2012;590(12):2917‐2936. 22495587 10.1113/jphysiol.2011.223800 PMC3448156

[49] Miyauchi S , Gopal E , Fei YJ , et al. Functional identification of SLC5A8, a tumor suppressor down‐regulated in colon cancer, as a Na^+^‐coupled transporter for short‐chain fatty acids. J Biol Chem. 2004;279:13293‐13296. 14966140 10.1074/jbc.C400059200

[50] Iwanaga T , Takebe K , Kato I , et al. Cellular expression of monocarboxylate transporters (MCT) in the digestive tract of mouse, rat, and humans, with special reference to slc5a8. Biomed Res. 2006;27:243‐254. 17099289 10.2220/biomedres.27.243

