# Supplementary figures and images for: Signaling of free fatty acid receptors 2 and 3 differs in colonic mucosa following selective agonism or coagonism by luminal propionate
Source: Neurogastroenterol Motil. 2018 Aug 23;30(12):e13454. doi: 10.1111/nmo.13454 (PMC6282569; doi:10.1111/nmo.13454)

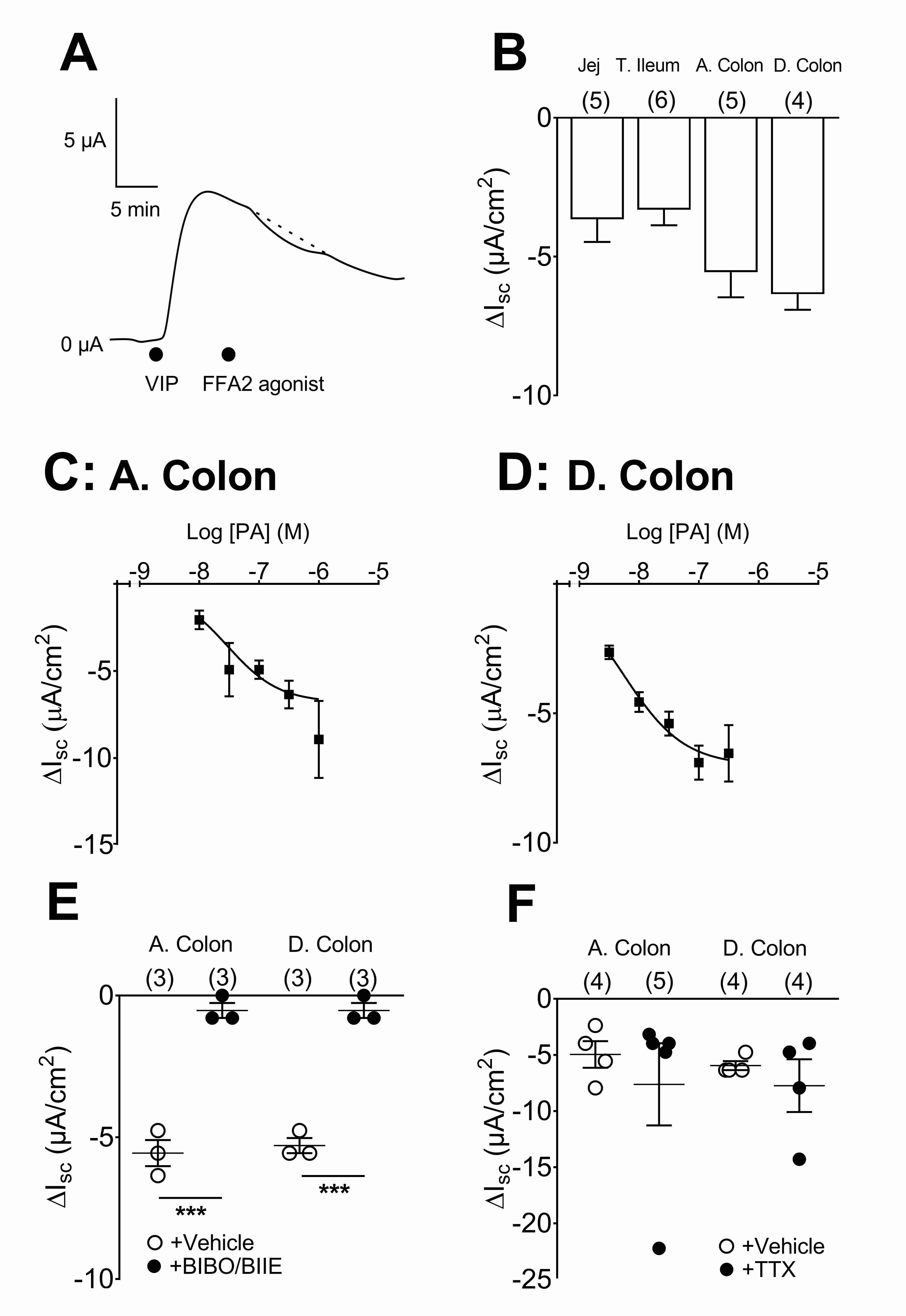

Supplement: Supplementary file 1 [file NMO-30-na-s001.jpg]
