# Supplementary material for: Signaling of free fatty acid receptors 2 and 3 differs in colonic mucosa following selective agonism or coagonism by luminal propionate
Source: Neurogastroenterol Motil. 2018 Aug 23;30(12):e13454. doi: 10.1111/nmo.13454 (PMC6282569; doi:10.1111/nmo.13454)
Supplement: Supplementary file 2 [file NMO-30-na-s002.docx]

**Supporting information**


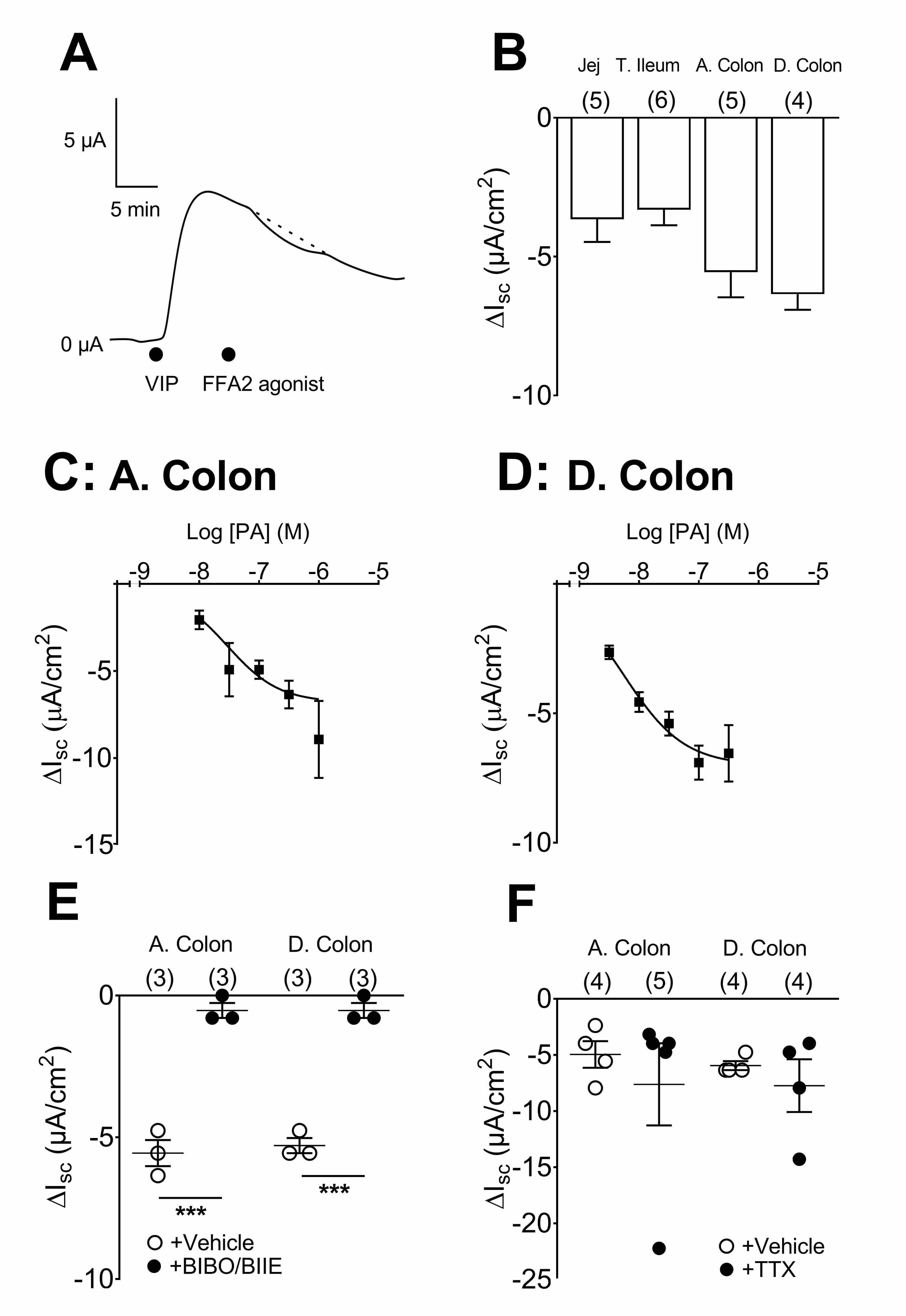


**Supplementary Figure 1.** Representative trace (A) showing FFA2-induced (PA, 100 nM apically) decreases in Isc after VIP pretreatment (30 nM) in mouse descending colon mucosa, and (B) a comparison of apical FFA2 agonism in mouse jejunum (Jej), terminal ileum (T. Ileum), ascending colon (A. Colon) and descending colon (D. Colon). (C) Concentration-response curves for apical PA, in A. colon and (D) D. colon mucosae. (E) Attenuation of apical FFA2 responses following Y1 and Y2 blockade with BIBO3304 and BIIE0246 (+BIBO/BIIE) in colonic mucosae, but not following TTX pre-treatment (F). Values are the mean ± 1SEM from 5-6 observations and statistical differences between control and experimental groups are shown (in E; *** *P* ≤ 0.001).


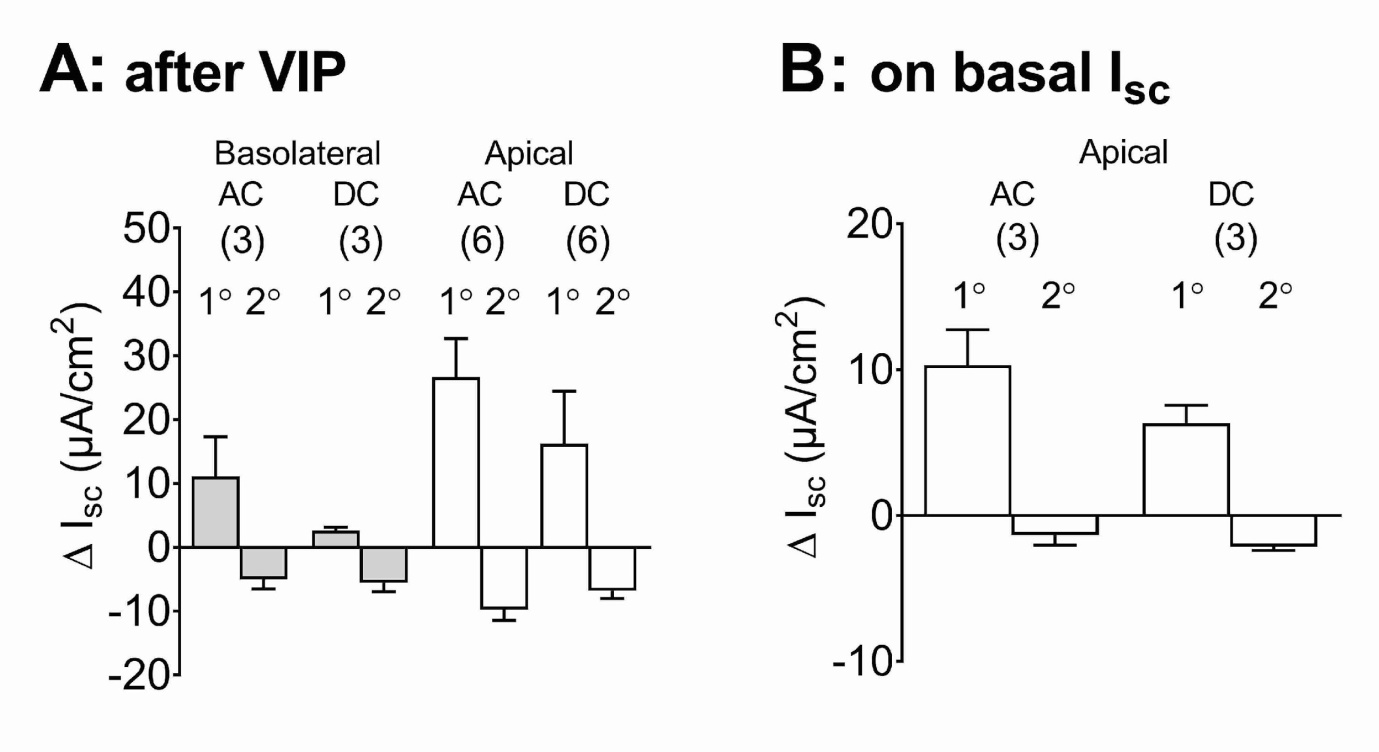


**Supplementary Figure 2.** Propionate (5 mM) elicits biphasic transport responses in mouse ascending colon (AC) and descending colon (DC) mucosae when added to the basolateral or apical reservoir in (A) after VIP (10 nM) pretreatment or, (B) on baseline Isc levels. Propionate responses comprised of an initial (1˚) increase in Isc followed by a slower (2˚) decrease in Isc in all cases. The 1˚ and 2˚ Isc components were each pooled and bars are the mean ± 1 SEM from the numbers of observations shown in parenthesis. There were no statistically significant differences.


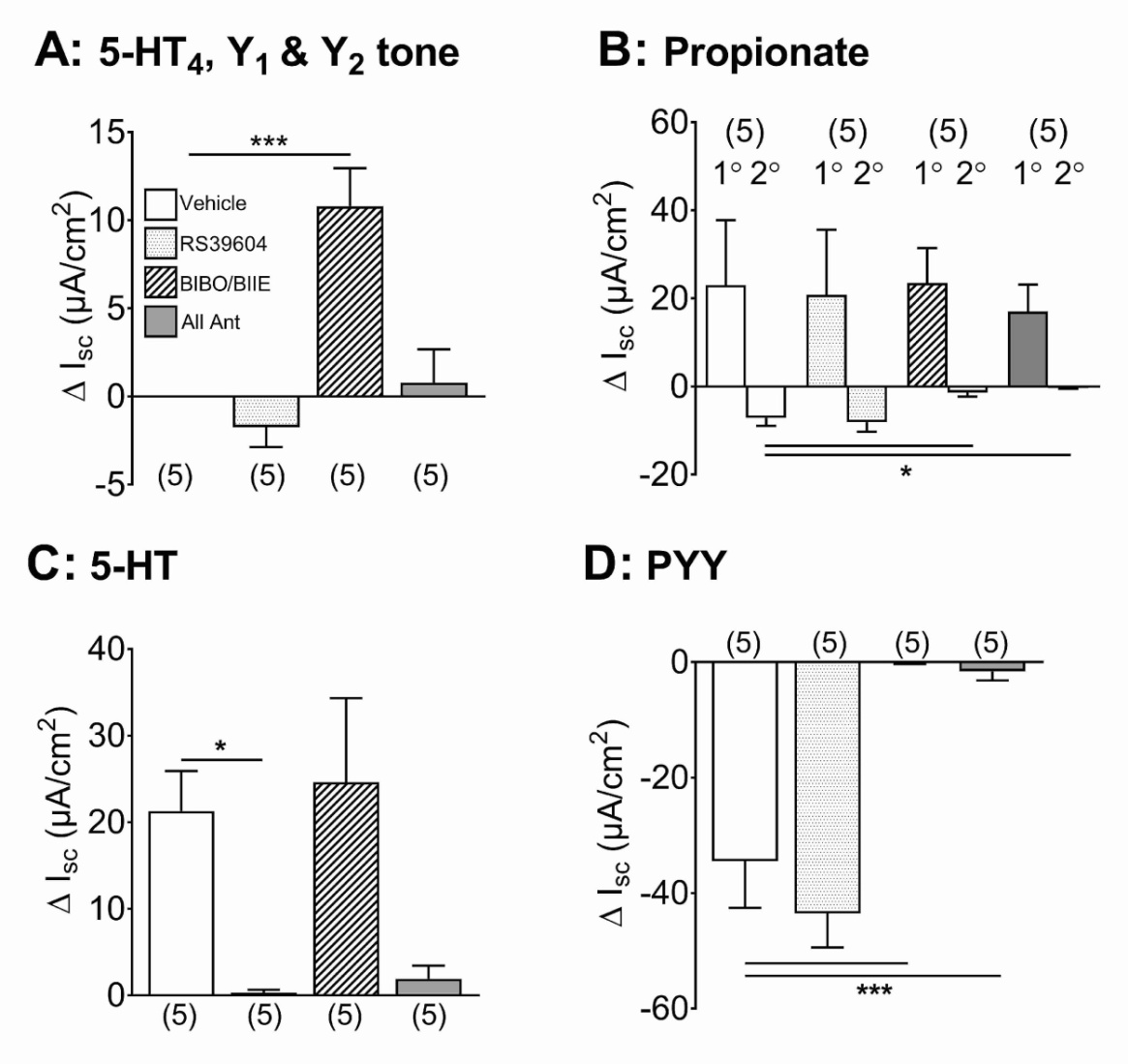


**Supplementary Figure 3.** Murine descending colon mucosal responses to (A) pretreatments with basolateral vehicle (H_2_O), 5-HT_4_ antagonist RS39604 (1 µM), a combination of the Y1 and Y2 antagonists (300 nM BIBO3304 and 1 µM BIIE0246; BIBO/BIIE) or all three blockers added together (All Ants). In (B) subsequent apical propionate (5 mM) responses were recorded after VIP (30 nM) addition. Control responses to (C) 5-HT (1 µM) and PYY (D: 10 nM) are shown. Bars are the mean ± 1 SEM from 5 colonic specimens, throughout.
